# Olfactory Obsessions: A Study of Prevalence and Phenomenology in the Course of Obsessive-Compulsive Disorder

**DOI:** 10.3390/jcm12093081

**Published:** 2023-04-24

**Authors:** Maciej Żerdziński, Marcin Burdzik, Roksana Żmuda, Paweł Dębski, Agnieszka Witkowska-Berek, Anita Pląder, Patrycja Mozdrzanowska, Marta Stawowy, Joanna Sztuk, Karolina Poremba, Magdalena Piegza, Piotr Gorczyca

**Affiliations:** 1 Faculty of Medicine, Academy of Silesia, 43rd Rolna Street, 40-555 Katowice, Poland; 2 Dr. Krzysztof Czuma’s Psychiatric Center, Psychiatric Department No 2, 27th Korczaka Street, 40-340 Katowice, Poland; 3 Institute of Legal Sciences and Doctoral School, University of Silesia, 12th Bankowa Street, 40-007 Katowice, Poland; 4 Department of Psychiatry, Faculty of Medical Sciences in Zabrze, Medical University of Silesia in Katowice, 49th Pyskowicka, 42-612 Tarnowskie Gory, Poland; 5 Mental Health Center, Medical Center ‘Femina’, 23th Kłodnicka Street, 40-703 Katowice, Poland

**Keywords:** OCD, olfactory obsession, sensory obsession, olfactory reference syndrome, OCRD, Olfactory Obsessions Questionnaire

## Abstract

Olfactory obsessions (OOs) are rarely described in the medical literature. The features of OOs appear consistent with characteristics of a typical obsession, but since they do not involve the realm of thought, it is questionable to term them obsessions per se. Olfactory Reference Syndrome (ORS) presents OOs inconsistently and is a distinctive diagnostic category related to OCD. Therefore, the primary objectives of our study were not only to assess the prevalence of OOs in OCD patients, but also to demonstrate their phenomenological consistency with other OCD symptoms. The study group consisted of 75 patients already diagnosed and treated for OCD. Hence, a comparison was made between OCD patients with and without OOs in terms of: symptom severity, level of insight and comorbidities. Olfactory obsessions (OOs) were found in 21.33% (*n* = 16). OOs induced compulsive behavior in more than 93% of subjects. The presence of OOs did not significantly differentiate the studied groups in terms of OCD severity (*p* = 0.876), level of insight (*p* = 0.680), depression (*p* = 0.746), mania (*p* = 0.525) and OCDP traits (*p* = 0.624). However, a comparison of the two groups showed that OOs patients presented higher levels of hostility (*p* = 0.036), cognitive impulsivity (*p* = 0.039), magic-type obsession (75% vs. 35.59%), and contamination obsession (87.50% vs. 67.80%). Conclusions: OOs frequently occur in the course of OCD, and their phenomenology is typical of this disorder. OOs are not a symptom of thought content disorders and are sensory in nature, which is not included in the definition of obsession. The presence of OOs in OCD provokes hostility and cognitive impulsivity. It can be assumed that the Olfactory Obsessions Questionnaire accurately identifies olfactory obsessions.

## 1. Introduction

Obsessive-compulsive disorder (OCD) is a syndrome of obsessions and compulsions. Obsessions are unwanted, intrusive and persistently recurring thoughts, urges or images, usually associated with particularly sensitive psychological areas of the sufferer (e.g., sexuality, religiosity or purity). Obsessions impair normal thought processes and in some cases make them extremely complicated or unsolvable; thus—causing overwhelming anxiety. Compulsions are repetitive physical or mental acts—ultimately avoidance behaviors—that the affected person feels forced to perform in response to the obsessions experienced. Compulsions involve simple activities (e.g., cleaning, checking or touching), but it can also occur as complicated and time-consuming rituals. OCD symptoms are usually identified by the patient as a problem, but it is also acceptable to diagnose OCD in the context of poor or lost insight [1,2,3,4].

Sensory obsessions, i.e., obsessions concerning the realm of the senses, in particular—the sense of smell (olfactory obsession, OOs)—appear to be a significant part of the varied symptomatology of OCD. It is worth noting that this phenomenon has not been more extensively described in the available medical literature, but in clinical practice it is not uncommon to encounter patients with OCD who, among other obsessions, report a sense of unpleasant and intrusive odor [5,6,7,8]. The existing description of OOs include features related to OCD: “The characteristics of olfactory obsession are exactly those of thought-related obsessions: the symptom is persistent, unwanted, and yet recurrent and unpleasant, uncontrolled by the patient’s will, associated with anxiety and leading to a corresponding compulsion that brings temporary relief” [5]. Ferrao et al. (2012) noted that a significant number of OCD patients report compulsions that are preceded not by obsessions, but by subjective experiences known as sensory phenomena [9]. The authors also suggested the need for continued research into the poorly understood problem of sensory symptoms in OCD [9,10]. OCD patients experiencing unpleasant odors were found to exhibit increased activation of the caudate nucleus and the left anterior and posterior cerebral hemispheres, which positively correlated with the severity of OCD symptoms, anxiety, frequency of disgust sensations and odor intensity ratings [11]. At this point, it is worth mentioning, that the Yale–Brown Obsessive Compulsive Scale (Y-BOCS), considered to be the primary diagnostic tool for OCD, does not contain questions that relate directly to the issue of OOs or obsessions concerning other sensory spheres [12,13].

Nozologically, olfactory obsessions appear to be most similar to Olfactory reference syndrome (ORS). ORS is characterized by a persistent belief that an unpleasant odor is emitted, even though objectively it is not perceptible. The most commonly reported sensations include clothing and body odor (sweat, urine or genitalia) or bad breath (halitosis). Occasional patients report emitting non-bodily odors such as detergent, ammonia, or onions [3,10,14,15,16,17]. The occurrence of OOs forces many repetitive behaviors: irrational sniffing, camouflaging or eliminating unpleasant odors through frequent bathing, washing, excessive use of cleaning products, air fresheners, etc. [5,16,18,19]. Feusner et al. (2010), in DSM 5 issues, described that individuals with ORS typically report disturbing, repetitive and intrusive thoughts about their odor, with which they are preoccupied for many hours each day [15]. They also note that some researchers [20,21,22] rightly refer to such thought preoccupation as obsessive thoughts, but further—they denied the concept that an intrusive smell is an obsession per se, which was supported by the thesis that OCD with delusional intensity affects less than 5% of patients, meanwhile, in the course of ORS, symptoms are more likely to have a delusional dimension. Yet, conceptualizing the definition of ORS, these authors propose identical levels of insight as those just occurring in OCD (good or fair insight, poor insight, delusional beliefs). The researchers noted that many ORS treatment case reports described improvement after serotonin reuptake inhibitor (SRI) monotherapy, and that some individuals did not respond to an antipsychotic drug, but again—responded specifically to SRI [23,24,25,26]. This findings again place ORS closer to OCD than to delusional disorders: SRIs, rather than antipsychotic treatment, are considered the most effective pharmacotherapy for OCD [27,28]. In order to diagnose ORS, it was recommended that schizophrenia and other psychotic disorders must be excluded, while the issue of differentiation from OCD was neglected [15].

Sensory symptoms have been described as frequently occurring not only in OCD, but also among other obsessive-compulsive symptoms in Tourette syndrome. It has been considered that sensory phenomena may be an important phenotypic indicator for grouping patients along the spectrum of OCD-Tourette’s disorder [8,29,30].

**Figure 1:**
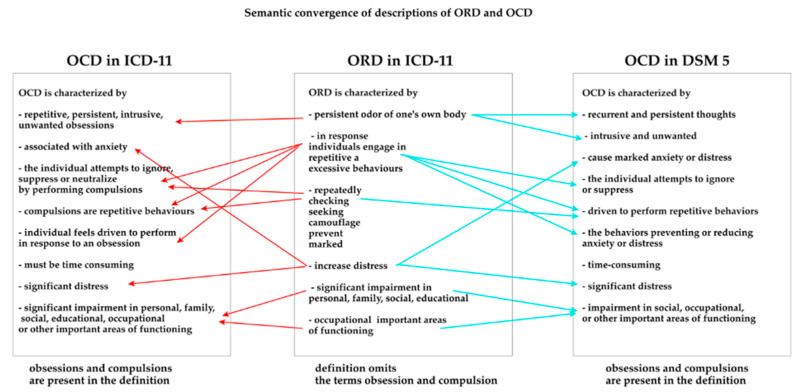
Semantic convergence of the ORD and OCD descriptions.

Olfactory phenomena were also regarded as persistent sensory delusional disorders (German: Sensitiver Beziehungswahn), which again distances them from OCD [15]. Both ORS and olfactory obsessions have been neglected in the ICD-10 classification [2]. In the expert guidelines to the ICD-11, Veale and Matsunaga (2014) suggest: “A co-occurring diagnosis of OCD is made only when obsessions are not limited to sniffing concerns” [19]. Finally, the ICD-11 assigned olfactory intrusions the title Olfactory Reference Disorder (ORD). It is defined as follows: “persistent preoccupation with the belief that one is emitting a perceived foul or offensive body odour or breath that is either unnoticeable or only slightly noticeable to others. Individuals experience excessive self-consciousness about the perceived odour, often with ideas of reference. In response to their preoccupation, individuals engage in repetitive and excessive behaviours such as repeatedly checking for body odour or checking the perceived source of the smell, or repeatedly seeking reassurance, excessive attempts to camouflage, alter, or prevent the perceived odour, or marked avoidance of social situations or triggers that increase distress about the perceived foul or offensive odour. The symptoms are sufficiently severe to result in significant distress or significant impairment in personal, family, social, educational, occupational or other important areas of functioning”. Although classified as an OCD-related disorder, the terms obsession/compulsion were not used. The diagnostic criteria only refer to body odor. The ICD-11 distinguishes three types of Olfactory reference syndrome: with fair to good insight (6B22.0), with poor to absent insight (6B22.1) and unspecified (6B22.Z). They are identical to the types of OCD [31]. DSM 5 did not address the issue of ORS as such. In this classification, olfactory symptoms are included in “Other specified OCD” as Jikoshu-kyofu (300.3). The olfactory symptoms here again concern only the fetor of one’s own body: “characterised by fear of having an offensive body odor” [1,3,10,32]. Figure 1 shows the significant semantic similarity of the ORD description compared to existing OCD definitions.

Considering the data cited above: descriptions of olfactory obsessions are not only relatively rare, but also do not refer to the term obsession per se (even as a subtype of OCD). Does the placement of different codes for symptoms so closely related have any practical dimension? If so, it is difficult for the authors of this study to determine its significance. This raises a fundamental question: how to accurately describe OO in consistency with other OCD symptoms? The purpose of our work is to answer this query.

## 2. Aims of the Study

The primary objectives of the study were to assess the prevalence of olfactory obsessions in patients with OCD and conduct a phenomenological analysis of OOs to demonstrate their coherence with other OCD symptoms.

The secondary objectives of the study included:

1. To compare two groups of patients (OCD with OOs and OCD without OOs) in terms of:
  - Basic sociodemographic data (age, gender, education, marital status);
  - Severity of OCD, the most common type of obsessions and compulsions occurring, and the level of insight;
  - Occurrence and severity of selected clinical phenomena considered common to OCD. These included: obsessive-compulsive personality (OCPD), depression (MDD), mania (M), aggression (A), and impulsiveness (I).
2. Analysis of subjects with OCD and OOs in terms of:
  - Assessing whether olfactory obsessions cause corresponding compulsions
  - Assessment the type of discomfort caused by olfactory obsessions.
3. To test the practical usefulness of the Olfactory Obsessions Questionnaire [33].

## 3. Materials and Methods

The study group was formed based on inclusion and exclusion criteria. The primary inclusion criterion was a diagnosis of OCD according to DSM 5 principles: the presence of obsessions and/or compulsions (criterion A) that consume a lot of time or cause significant distress or impairment in the functioning of the individual (criterion B). Investigators assessed OCD criteria based on personal interview with the patient and the treatment records provided. In order not to bias the assessment of the clinical picture of OCD, patients with severe affective symptoms were excluded from the study. The following values were assumed: ≥30 scores on the Hamilton Depression Scale and ≥37 scores on the Young Mania Rating Scale. It also ensured that all subjects were able to clearly understand the objectives of the study. Patients with a history of epilepsy were also not included (to exclude uncinated fits during which olfactory hallucinations occur) [34]. Eighty-two patients with OCD were initially invited to participate in the study, based on a list that was compiled from subjects previously diagnosed and treated for this condition. Seven patients were excluded due to above-mentioned exclusion criteria (six due to very severe depression, one due to severe mania, none due to epilepsy). To avoid biasing the results, all subjects excluded from the study were not previously assigned to either of the two study groups. Therefore, diagnosis for olfactory obsessions was not initiated in these patients. Finally, the study was conducted in a group of seventy-five patients with OCD. Of the total number of participants, sixty-six were examined in an outpatient setting and nine in a psychiatric ward. An informed consent was obtained from each patient after clarifying the purpose, nature and procedures of the study. The study caused no additional burden for the patients: apart from its diagnostic value, it was also psychoeducational in nature. All investigators were medical professionals (psychiatrists, psychologists and psychiatric nurse) employed in centers that diagnose and treat mental disorders. The study was conducted in one site (Dr. Krzysztof Czuma’s Psychiatric Center), with the substantive cooperation of medics from all aforementioned psychiatric centers. Each of the investigators was trained for all the diagnostic tools used. The obtained results were each time assessed simultaneously by at least two investigators to ensure the reliability of the research. The local bioethics committee has approved the study.

The subjects were divided into two groups: group 1—patients with OCD and with OOs, group 2—patients with OCD but without OOs. Both groups were subsequently compared in terms of sociodemographic data. We also assessed and compared: severity of OCD, type of most common obsessions and compulsions, the level of insight, the number of OCPD traits, the current level of aggression and its components (physical aggression, verbal aggression, anger, hostility), the current level of impulsivity and its components (cognitive, motor and unplanned impulsivity), and the current severity of a depression and mania. All the phenomena mentioned are considered common in the course of OCD [35,36,37,38,39]; therefore, it was interesting to conduct a comparison of their occurrence to find potential differences in the both study groups.

The OCD with OOs group was also assessed whether olfactory obsessions cause corresponding olfactory compulsions, as well as whether they induce discomfort (if so, what type).

The following diagnostic tools were used:

- The Yale–Brown Obsessive Compulsive Scale (Y-BOCS). The Y-BOCS is commonly considered the “gold standard” for assessing the presence, severity and monitoring of OCD symptoms [10]. The first part of the scale is intended to identify OCD symptoms (obsessions and compulsions). The second part measures five parameters of OCD: duration of symptoms, level of dysfunction and distress caused by symptoms, ability to resist symptoms and ability to control. Each parameter is rated on a 5-point scale (0–4). Total score (0 to 40) indicates OCD severity, with a score of 0–7 indicating subclinical symptoms, 8–15 mild symptoms, 16–23 moderate symptoms, 24–31 severe symptoms and 32–40 extreme symptoms [40].
- Olfactory Obsessions Questionnaire (OOQ). The tool was developed by Żerdziński (1998). The questionnaire is intended to diagnose olfactory obsessions and to analyze their phenomenology. The questions relate to the self-identification of OOs, as well as the nature of their perception (not only the sense of smell is taken into account), the level of insight, the type of emotions evoked (irritability, anxiety, distress, mental tension, embarrassment) and the type of co-occurring compulsions resulting from OOs (mental compulsions are also taken into consideration). Finally, OOQ scores are not metrically assessed, but are easily interpreted by the clinician; therefore, they can be helpful in diagnostic and clinical work with OCD patients [5,33] (Appendix A).
- Hamilton Depression-Rating Scale (HDRS-17). HDRS is a tool used to diagnose depressive disorder. It contains 21 items, each rated on a 5-point scale (0–4). The total score is used to assess the severity of depression (<7—no depression; 8–16—mild depression, 17–23—moderate depression; 18 to 29—severe depression, ≥30—very severe depression) [31,41].
- Young Mania Rating Scale (YMRS). YMRS is an 11-item tool to assess manic symptoms. The answers are rated on a 5-point scale (0–4), except for 4 items, which are scored 0–8. The total score of ≥38 indicates severe mania [42].
- Buss-Perry Aggression Questionnaire (BPAQ). This 29-item tool is used to assess the level of aggression and its components: physical aggression (PA)—9 items; verbal aggression (VA)—5 items; anger (A)—7 items; hostility (H)—8 items [43].
- Barratt Impulsiveness Scale (BIS-11): a 30-item self-report questionnaire to assess impulsiveness based on the frequency of a given behavior (rarely/never, occasionally, often or almost always). It allows to measure the three theoretical subtraits of impulsiveness, i.e., cognitive, motor, and non-planning impulsiveness [44,45].
- Brown Assessment of Belief Scale© (BABS). The BABS scale has been developed to rate the degree of conviction and insight patients have concerning their beliefs. BABS rates a number of dimensions that underlie delusional and nondelusional beliefs: conviction, perception of others’ views of beliefs, explanation of differing views, fixity of ideas, attempt to disprove beliefs, insight, and ideas/delusions [46].
- The presence of OCPD was assessed according to DSM 5 criteria. Five or more features (out of a total of eight) were considered significant for diagnosis [1]. In this study, we only assessed the number of OCPD traits (anacasticity level).

Statistical analyses were performed using Excel 2016 and Statistica version 13.3. Shapiro-Wilk test was used to assess the normality of the distribution of variables. The significance of differences in continuous variables between the group of OCD patients with OOs and the group of OCD patients without OOs was assessed with the Mann-Whitney U test. The correlations between the individual obsessions and compulsions in both subgroups were assessed with the chi-square test with Yates’ correction. The effect size measure was calculated using Cohen’s d coefficient. Statistical significance was set at α ≤ 0.05.

## 4. Results

Our results confirmed that olfactory obsessions are common in the course of OCD. They were found in sixteen of the overall seventy-five subjects (21.33%) (group 1). Fifty-nine individuals (78.67%) did not experience OOs (group 2). Basic sociodemographic results for study groups are presented in Table 1.

**Figure 2:**
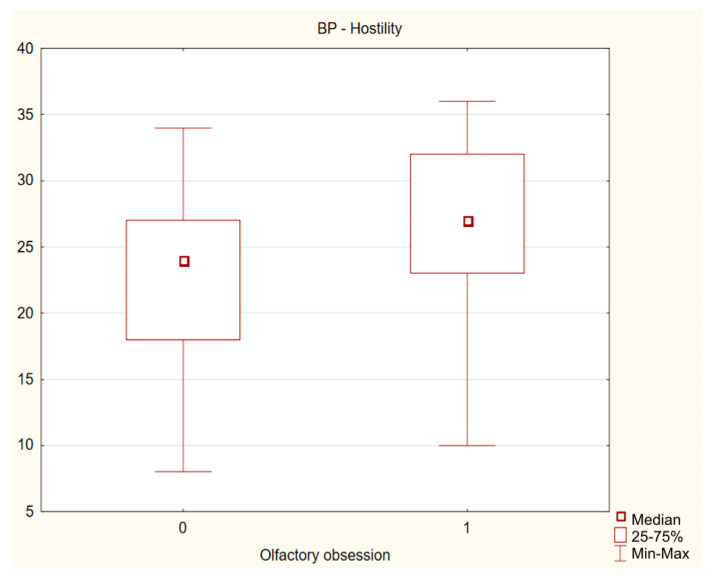
Difference in severity of hostility between OCD patients with and without olfactory obsessions.

**Figure 3:**
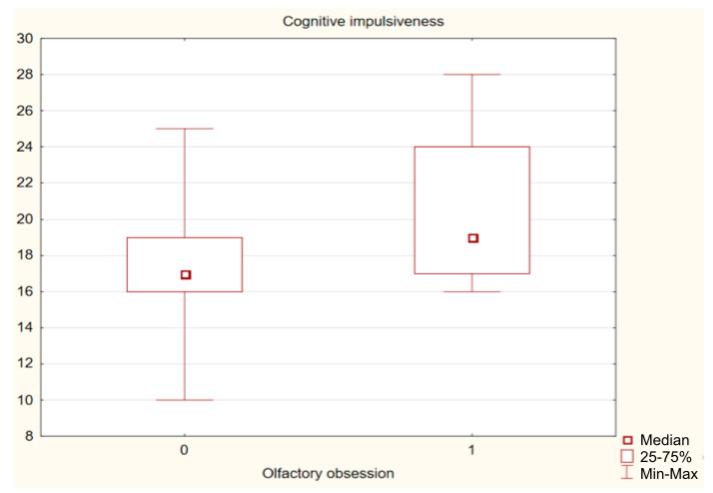
Difference in severity of cognitive impulsivity between OCD patients with and without olfactory obsessions.

A comparison of the total group and both subgroups showed that the presence of OO did not significantly differentiate patients in terms of the basic criteria of OCD: severity of symptoms and level of insight. No significant differences were found between the study groups for the severity of affective symptoms: depression (*p* = 0.746) and mania (*p* = 0.525). The number of OCDP traits, the level of generalized aggression and general impulsivity were also similar. Significantly higher levels of hostility (BP-H) and cognitive impulsivity (Imp cog) were reported by OCD patients with OO. The effect size measured with Cohen’s d coefficient indicates that the size of possible differences between groups is negligible. The results are presented in Table 2 and Table 3 and Figure 2 and Figure 3.

**Figure 4:**
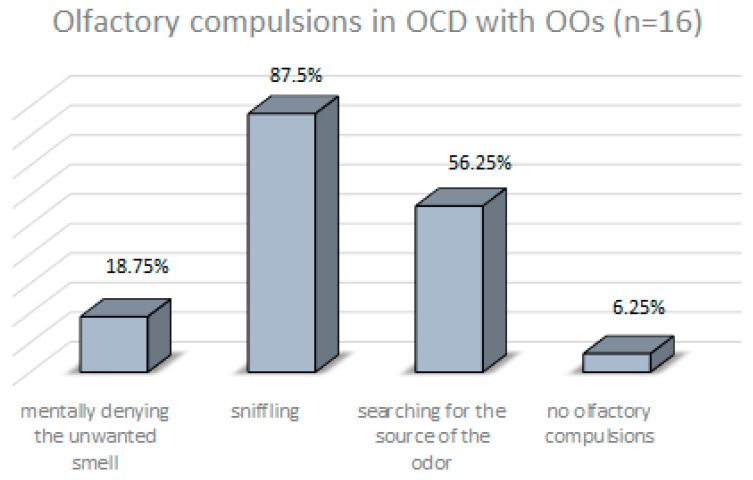
Types of compulsions in response to olfactory obsessions in patients with OCD and OOs.

The vast majority of subjects in group with OOs (87.5%) reported that olfactory obsession caused discomfort (distress), which was most often associated with irritability (68.75%), anxiety (62.5%), mental tension (50%) and embarrassment (25%). One person (6.25%) was unable to specify the emotions caused by OOs. Olfactory obsessions were most often related to body/clothing odors (50%), odors of organic secretions (37.5%) and odors of chemicals (31.25%). Among others, food (25%), dirt (12.5%), animals (6.25%) and smoke (6.25%) were identified. The most common compulsions studied in response to olfactory obsessions are shown in Figure 4. It is worth noting that compulsions associated with OOs did not occur in the group of patients without OOs (*n* = 59, olfactory compulsions = 0).

**Figure 5:**
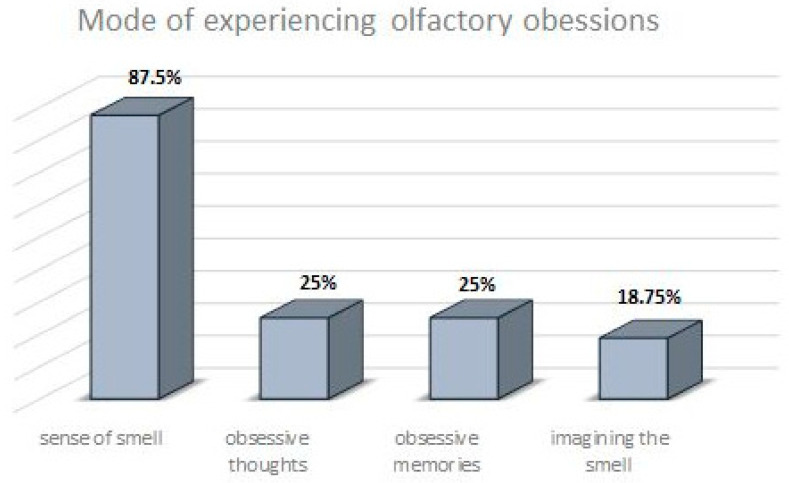
The most commonly reported modes of experiencing olfactory obsessions in patients with OCD and OOs.

87.5% subjects of OCD with OOs reported that they experience OOs primarily through the sense of smell, and 62.5% respondents answered that this is the only modality in which they perceive these symptoms (OOQ item 4: “Sniffing that smell”). The odor experience was also associated with obsessive thoughts (25%), obsessive memories (25%) or images (18.75%). Two patients considered olfactory obsessions to be experienced in thought and without sensory recognition per se (response in OOQ item 4: “Thinking about this smell”—1; “Remembering this smell”—1, respectively). These findings are shown in Figure 5.

**Figure 6:**
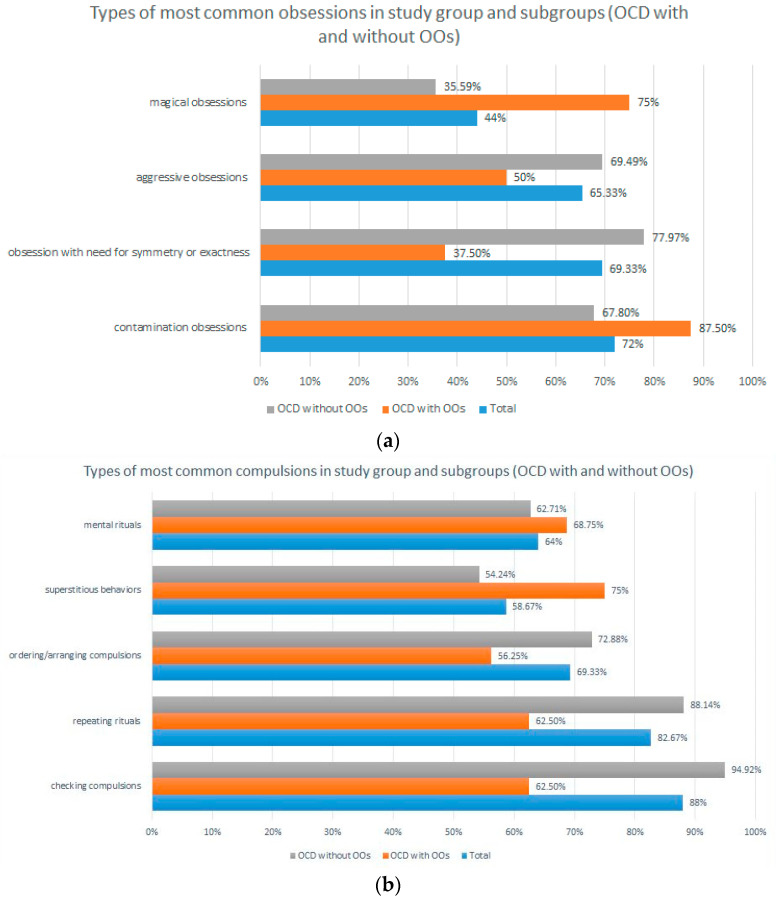
(**a**) Prevalence of the most common obsessions in the overall study group and in the two study subgroups (OCD with and without OO). (**b**) Prevalence of the most common compulsions in the overall study group and in the two study subgroups (OCD with and without OO).

The results for the most common obsessions among the total study group were: contamination obsessions (72%), obsession with need for symmetry or exactness (69.33%), and aggressive obsessions (65.33%). The most common compulsions were: checking compulsions (88%), repeating rituals (82.67%), and ordering/arranging compulsions (69.33%). In the group with OOs, the most frequently observed obsessions were: contamination obsessions (87.50%) and magical obsessions (75%). The most common compulsions were: superstitious behaviors (75%), mental rituals (68.75%), checking compulsions (62.50%) and repeating rituals (62.50%). Correspondingly, the most common obsessions in the group without OOs were: obsession with need for symmetry or exactness (77.97%), aggressive obsessions (69.49%), and contamination obsessions (67.80%). In terms of compulsions: checking compulsions (94.92%), repeating rituals (88.14%), ordering/arranging compulsions and (72.88%), and mental rituals (62.71%). In a comparison of the two study groups, patients with OOs were more likely to experience magic-type obsessions, while this type of obsession was less common in those without OOs (chi2 = 7.933; *p* = 0.005). A lower prevalence of obsessions related to the need for symmetry and order was observed among those with OOs. In subjects without OOs, the presence of such obsessions was significantly more frequent (chi2 = 9.694; *p* = 0.004). The results are shown in Figure 6a,b.

## 5. Discussion

As mentioned in the introduction, olfactory obsession is not included in any current or past definition of OCD and is rarely and inconsistently described in the medical literature. ORS/ORD is a valuable attempt to characterize OOs and accurately presents obsessive-compulsive phenomena involving the sense of smell. However, ORS/ORD peculiarly camouflages co-occurring compulsions by describing them with other words: “repetitive behaviors”. Since anankastic terms are not used to describe ORS per se, this is perhaps where the idea of creating more OCD-related categories (in both ICD-11 and DSM 5) derived from. In this regard, we have some concerns about whether the multiplication of diagnostic sub-categories is the most accurate direction for further researches into OCD issues. Therefore, it was interesting to conduct a study on OOs, subsequently define this symptom and further—to assess whether the presence of OOs significantly differentiates patients with OCD. If our study showed significant phenomenological differences of OOs relative to typical obsession, the validity of distinguishing the olfactory subtype of OCD could be confirmed. It would also be possible to hypothesize that OOs is not an obsession per se. Our results rather suggest expanding the definition of OCD to include the olfactory aspect.

**Figure 7:**
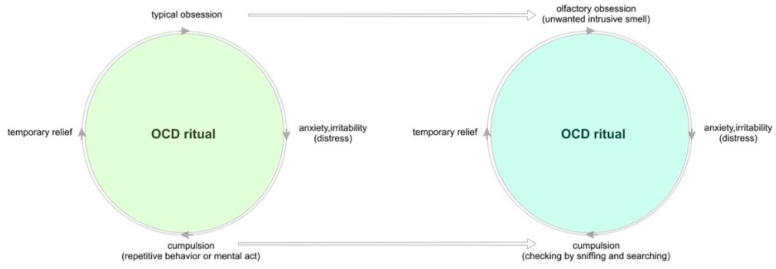
Comparison of pathomechanisms in typical OCD and OCD with olfactory obsessions.

Olfactory obsessions occurred in sixteen of the seventy-five OCD patients assessed, which can be considered common (21.33%). We also confirmed the primary research hypothesis: OOs are not phenomenologically different from other obsessions. Thus, OOs share all the traits of a typical obsession: they are unwanted, intrusive and persistently recurring. They also cause such severe discomfort (87.5% of patients with OCD and OOs) that compulsive behaviors are the only method of obtaining temporary relief (93.75% of OO patients), which corresponds to the cited observations of Żerdziński (2008) and Ferrao (2012) [5,9]. It is relevant that the compulsions found in the group with OOs (sniffing, searching for the source of the odor, mentally denying the unwanted odor) were very closely related to olfactory obsessions and did not occur in those without OOs (100% vs. 0%). In addition to the metric scores, all investigators also monitored patients’ emotional reactions while completing the OOQ. Every subject experiencing OOs considered these symptoms to be an integral part of the clinical picture of OCD and showed no surprise or doubt about the diagnostic questions on the OOQ form. Figure 7 shows the identity of the pathomechanisms occurring in OCD with OOs and in typical OCD.

Equally interesting is the analysis of the results regarding the characteristics of OOs. The dominance of obsessions concerning body and/or clothing odors and organic secretions (50% and 37.5%, respectively) may be related to the psychological functions of OCD, among which low self-esteem and self-harm are considered common and therapeutically relevant [46,47]. However, olfactory obsessions involved more than just own body odor. Respondents also indicated sensation of unpleasant smells of chemicals (31.25%), food (25%), dirt (12.5%), animals (6.25%) and smoke (6.25%). These results demonstrate the unnecessary restriction of olfactory phenomena to symptoms related to the body’s own fetor, as suggested in the ORS [10,14,15,16]. Since it is acceptable to diagnose thematically diverse thought obsessions [1,2,3,4], the definitions of ORS and ORD should also allow for many other sources of odor.

It is worth discussing the phenomenology of OOs in the context of existing definitions of OCD. While OOs-related compulsions have characteristics typical of other compulsions found in OCD without OOs (“repetitive behaviors or mental acts that the individual feels driven to perform in response to an obsession or according to rules that must be applied rigidly”) [1,2,3,4], defining the concept of olfactory obsession is much more difficult. Obsessions have been defined as: ‘recurring and persistent thoughts, urges or impulses (…)’ [1,2,3,4]. The vast majority of our patients (87.5%) experienced OOs through the sense of smell, which refers to the sensory modality rather than the context of thoughts, urges or impulses. Thus, classifying OOs as a thoughts disorder seems inaccurate. According to this approach, we can only consider the concept of memory obsession with a particular odor, which, however, is a physiologically borderline theory and questionable in terms of objective nosological categorization. It was not confirmed in our study: odor memory verified by OOQ as the only to experience olfactory obsession was noticed in one study case. Therefore, our results encourage to expand the definition of obsession to include the olfactory context. This thesis appears to be indirectly supported by recent classification changes, which, as mentioned in the introduction, place ORS closer to OCD than delusional disorders [1,3,32].

As mentioned in the introduction, OOs has been also missed in the diagnostic questions of all commonly used diagnostic scales for OCD (including Y-BOCS and Maudsley Scale) [48]. Therefore, we used the Olfactory Obsessions Questionnaire, the only tool dedicated to the diagnosis of OOs that has been described in the medical literature [33]. The OOQ did not provoke any objections from either the researchers or the subjects themselves, allowing us to accurately identify and then analyze olfactory obsessions. Our further research on olfactory OCD will aim to prove the psychometric effectiveness of the Olfactory Obsessions Questionnaire.

The both study groups were similar in terms of basic sociodemographic parameters, ensuring a fair comparison. It was found that subjects with and without OCD did not differ significantly in the severity of OCD. The results for level of insight and number of OCPD traits were also similar. There were no significant differences in the severity of depression, mania, anger (except for hostility) and impulsiveness (except for cognitive impulsiveness). It should be recalled that seven subjects were excluded from the study due to the presence of severe and potentially psychotic affective symptoms that may have caused inaccurate responses. From depressive guilt at a potentially delusional level, or from inflated good mood and grandiosity delusions, these patients may have manifested a morbid desire to meet all the expectations of our study. Thus, these individuals may have confirmed symptoms that they are not actually experiencing—in this case, OOs. While the severity of OCD was similar in both study groups, the results regarding the prevalence of individual obsessions and compulsions were different. Contamination and magic obsessions were more common in the OCD with OOs (contamination obsessions—87.50% vs. 67.80%; magic obsessions—75% vs. 35.59%). In contrast, aggressive and order/symmetry-related obsessions were more frequent in the OCD group without OOs (aggressive obsessions—69.49% vs. 50%; order/symmetry-related obsessions—77.97% vs. 37.50%). The results regarding compulsions are also interesting. In the group without OOs, compulsions of checking and repeating rituals occurred more often (compulsions of checking: 94.92% vs. 62.50%; repeating rituals: 88.14% vs. 62.50%), while superstitious behaviors were more common in the group with OOs (75% vs. 54.24%).

The differences presented in the results may be due to the relatively small subgroups of patients, but they are worth interpreting in psychological terms, which may be useful in OCD psychotherapy. Were the contaminations and magic-type obsessions as well as superstitious behaviors found in the OCD-OOs group specifically transferred to the sense of smell? It is assumed that sniffing is a motor link of human affective processes that allows an individual to adapt to environmental conditions or events by displaying adapted behavior [49]. Therefore, the presence of olfactory obsession in the course of OCD may indicate distrust of the environment. Our hypothesis is supported by the results of other researchers: distrust (which ultimately provokes hostility) is a typical feature of OCD patients [35]. Sookman et al. (2001) confirmed that the need for control in OCD patients was higher than in other clinical groups and in healthy individuals [50]. Another study reports a link between locus of control (LOC), beliefs about the importance and control of thoughts, and general OCD symptoms [51]. It has been also described that compulsive behaviors (such as checking) cause a reduction in anxiety, resulting in a sense of regaining control over the environment [52]. The results of our study showed that patients with OCD and OOs present a strong need for control and this may have transferred to the area of olfactory obsessions. OOs caused such strong discomfort (87.5%) that compulsive behavior was the only method of obtaining temporary relief (93.75%). In response to olfactory intrusions, the most common type of compulsion was actually sniffing (occurring in as many as 87.5% of patients with OOs), an activity that directly involved the sense of smell.

## 6. Conclusions

Olfactory obsessions are common in the course of OCD and phenomenologically do not differ from “typical” obsessions. Olfactory obsessions involve a variety of smells, not just your own body odor. The occurring of olfactory obsessions causes severe discomfort and corresponding compulsions. Olfactory obsessions are not a symptom of thought content disorders: they are sensory in nature. It is worth expanding the definition of obsession to include an aspect related to the sense of smell. The occurrence of olfactory obsessions in OCD is associated with higher levels of magical and contamination obsessions, and with superstitious behaviors. The presence of olfactory obsessions provokes hostility and cognitive impulsivity. It can be assumed that the Olfactory Obsessions Questionnaire accurately identifies olfactory obsessions. Psychometric validation of this questionnaire is therefore worthwhile. Olfactory phenomena present a poorly understood psychopathological problem in OCD that deserves further study.

## 7. Limitations of the Study

This study had some limitations:

1. A relatively small sample of patients with OCD (*n* = 75) allowed for the identification of only 16 patients with OOs. The small sample of OCD subjects may have biased the results of the study, and thus the discussion and conclusions provided. Considering the above, the results of studies conducted on a larger population of patients with olfactory obsessions may be different.
2. The lack of diagnostic tools for olfactory obsession other than the OOQ may have disturbed the objectivity of the results obtained, especially since a psychometric evaluation of the Olfactory Obsession Questionnaire has not yet been conducted.
3. Only selected disorders and phenomena were included in the comparison of comorbidities. It is possible that there are others factors, the evaluation of which could shape the obtained results differently.
4. More than 93% of the subjects received drug treatment. Therefore, the analyzed correlations may shape differently in groups of OCD who do not receive any medication. Analysis of this aspect requires further research due to the small number of non-drug-taking patients (<7%) in this study.
5. Due to the preliminary nature of the study, exogenous factors that may affect the sense of smell (such as smoking nicotine) were not included in the study. This issue will be analyzed in further research.
6. It is difficult to assess whether the exclusion criterion of patients with severe and potentially psychotic depression—even before testing for olfactory obsessions—may have affected the results of the study.

## Figures and Tables

**Table 1:** Sociodemographic characteristics of OCD patients with and without olfactory obsessions.

Group		Study Group (Total) *n* = 75	Group 1 (OCD with OOs), *n* = 16	Group 2 (OCD without OOs), *n* = 59
age		Mean 45.12 yrs.; SD 12.43	Mean 42.06 yrs.; SD 10.11	Mean 45.95 yrs.; SD 12.86
gender	females	40 (53.33%)	7 (43.75%)	33 (55.93%)
	males	35 (46.67%)	9 (56.25%)	26 (44.07%)
education	less than higher	31 (41.33%)	8 (50%)	23 (38.98%)
	higher	44 (58.67%)	8 (50%)	36 (61.02%)
marital status	relationship (formal or informal)	48 (64%)	10 (62.5%)	38 (64.41%)
	single	27 (36%)	6 (37.5%)	21 (35.59%)

**Table 2:** Descriptive results on OCD severity and level of insight in patients with and without olfactory obsessions.

		OCD Total (*n* = 75)	OCD with Olfactory Obsessions (*n* = 16)	OCD without Olfactory Obsessions (*n* = 59)
OCD-severity	Mild	15 (20%)	3 (18.75%)	2 (20.34%)
	Moderate	22 (29.33%)	4 (25%)	18 (30.51%)
	Severe and extremely severe	38 (40.67%)	9 (56.25%)	29 (49.15%)
Insight	Good/fair	53 (70.67%)	12 (75%)	41 (69.49%)
	Poor and lost	22 (29.33%)	4 (25%)	18 (30.51%)

**Table 3:** Comparison of phenomena occurring in OCD patients with and without olfactory obsessions.

	OCD without Olfactory Obsessions (*n* = 59)			OCD with Olfactory Obsessions (*n* = 16)			Mann-Whitney U Test		Cohen’s d
	Mean	SD	Median	Mean	SD	Median	Z	*p*	
Y-BOCS	22.932	7.416	23.000	22.250	6.319	24.500	0.155	0.876	0.013
BABS	1.458	1.039	2.000	1.625	1.088	1.500	−0.413	0.680	−0.006
OCPD	3.780	2.126	4.000	4.250	2.380	4.000	−0.490	0.624	−0.097
BP Scale	78.000	17.843	78.000	81.875	13.291	82.000	−0.660	0.509	−0.013
BP-PA	18.271	6.233	17.000	16.938	5.662	15.500	0.831	0.406	0.035
BP-VA	14.475	4.348	14.000	15.438	3.596	15.000	−0.947	0.343	−0.053
BP-A	21.627	5.863	22.000	22.625	3.739	23.000	−0.382	0.702	−0.031
BP-H	23.051	6.358	24.000	26.750	6.517	27.000	−2.100	0.036 *	−0.089
Imp	62.508	9.916	62.000	67.000	14.000	61.000	−0.731	0.464	−0.035
Imp cog	17.542	3.390	17.000	20.250	3.975	19.000	−2.061	0.039 *	−0.213
Imp motor	20.475	4.591	20.000	22.813	5.504	21.500	−1.560	0.119	−0.100
Imp plan	24.508	5.920	24.000	22.688	5.069	23.000	0.856	0.392	0.054
HDRS	8.542	5.891	8.000	9.500	7.174	7.500	−0.324	0.746	−0.061
YMRS	3.475	4.329	2.000	4.875	5.807	3.000	−0.636	0.525	−0.024

Note: Y-BOCS—general severity of OCD, BABS—insight into the disease, OCPD—number of anankastic personality traits, BP Scale—Buss-Perry Scale general score, BP-PA—physical aggression, BP-VA—verbal aggression, BP-A—anger, BP-H—hostility, Imp—impulsiveness general score, Imp cog—cognitive impulsiveness, Imp motor—motor impulsiveness, Imp plan—planning impulsiveness, HDRS—Hamilton Depression Rating Scale, YMRS—Young Mania Rating Scale, *—statistically significant *p* ≤ 0.05.

## Data Availability

The data presented in this study are available on request from the corresponding author.
